# What’s new in minimally invasive thoracic surgery? Clinical application of augmented reality and learning opportunities in surgical simulation

**DOI:** 10.3389/fsurg.2023.1254039

**Published:** 2023-11-02

**Authors:** Ilaria Onorati, Dana Mihaela Radu, Emmanuel Martinod

**Affiliations:** ^1^ Chirurgie Thoracique et Vasculaire, Assistance Publique – Hôpitaux de Paris, Hôpitaux Universitaires Paris Seine-Saint-Denis, Hôpital Avicenne, Université Sorbonne Paris Nord, Faculté de Médecine SMBH, Bobigny, France; ^2^ Inserm UMR1272, Hypoxie et Poumon, Université Sorbonne Paris Nord, Faculté de Médecine SMBH, Bobigny, France

**Keywords:** augmented reality, NSCLC, mixed reality, surgical simulation and training, VATS

## Abstract

Lung cancer represents the most lethal cancer worldwide. Surgery is the treatment of choice for early-stage non-small cell lung cancer, with an overall survival that can reach 90% at 5 years, but its detection is difficult to achieve due to the lack of symptoms. Screening programs are crucial to identify small cancer. Minimally invasive surgery has modified the therapeutical approach of these tumors, becoming the standard of care, with an important clinical yield in terms of reduction of postoperative pain and length of hospital stay. The aim of this mini-review is to explore and describe two important and innovative aspects in the context of “growing opportunities in minimally invasive thoracic surgery”: the clinical application of augmented reality and its advantages for patient and surgeon, and the pedagogical issue through simulation-based training.

## Introduction

One in five people worldwide develop cancer during their lifetime. Early diagnosis is the key to reducing cancer-related mortality. Prevention represents one of the most significant public health challenges of the 21st century (1). Lung cancer represents the second in terms of incidence and the most lethal cancer worldwide (2). Accurate understanding of social characteristics of health is critical in identifying limits of screening of all populations (3).

Lung cancer has a generally poor prognosis, with an overall 5-year survival rate of 20.5% (4). However, early-stage lung cancer has a better prognosis and is more responsive to treatment, but its detection is difficult to obtain due to the lack of symptoms. In this context, appropriate screening is crucial. Low-dose computed tomography (LDCT) has high sensitivity and specificity for the detection of lung cancer, with demonstrated benefit in screening high risk patients (4–6). In near future, artificial intelligence will probably improve lung cancer screening procedures by a standardized and personalized evaluation and less irradiation needed to get performing imaging (7).

Surgery is the treatment of choice for early-stage lung cancer. The success and development of the minimally invasive approach has seamlessly integrated the treatment of these tumors to become the standard of care. The aim of this mini-review is to explore and describe two important and innovating concepts in “growing opportunities in minimally invasive thoracic surgery”: the clinical application of augmented reality and its advantages for patient and surgeon, and the pedagogical issues of these techniques for trainees by simulation-based training. Minimally invasive approaches involve both video-assisted and robotic-assisted but in this mini-review we have chosen to treat exclusively the video-assisted thoracoscopic surgery (VATS) approach.

## VATS and technology

Minimally invasive techniques have taken the forefront in thoracic surgery for the treatment of early-stage lung cancer. Different approaches have been described as “minimally invasive”. The common point between these techniques is that they are non-rib spreading thoracic procedures. VATS has been the first described. The era of VATS dates back to the 80s but its full development would come only 20 years later thanks to the advances in technology applied to surgical support materials. Ever since, the industry has been focused not only on the improvement and development of adapted surgical instruments, but also on providing tools for intraoperative surgical guidance and lesion detection. The interest in the use of technology in surgery has only increased, to the point of merging through the application of augmented and virtual reality and artificial intelligence. Surgical techniques have also improved, and VATS is now the standard procedure for early-stage NSCLC.

Different VATS techniques have been described, differing in type of approach, instruments and number of trocars. Each technique represents a “philosophy” of the way of doing surgery. From the multiport to the uniportal approaches, the advantages of VATS have largely been described in terms of less postoperative pain, reduction of drainage time and length of hospital stay, increased delivery of adjuvant therapy, less consequence on pulmonary function tests and the immune system, better outcomes, and a global positive impact in quality of life (8).

## Which cancer to be treated by VATS?

Screening programs and, more recently, radiological follow-up of COVID-19 pneumonia allowed for the detection of early clinical stage IA, small-sized NSCLC. Lobectomy is the standard care for early stage NSCLC (T1-2a, N0, M0) and, for more than 25 years has been considered the best surgical treatment and VATS has played an important role in the treatment of these patients in the last decade. However, dealing with small cancers, incited the medical community to consider the study of sublobar anatomical resections as the treatment of choice in patients with tumor size <2 cm. Recent trials have shown the non-inferiority of segmentectomy vs. lobectomy in these cases in terms of overall survival (9).

## VATS segmentectomy: a new challenge for the thoracic surgeon?

Segmentectomy is thus considered a valid alternative to lobectomy in terms of cancer radicality (10), preservation of lung functions, but it represents a more technically challenging procedure compared to lobectomy. It started to be widely performed instead of lobectomy for peripheral small-sized non-small cell lung cancer (11) and its use would be expected to increase in the near future. With the current tendance that “less is more” (11) we must not forget that surgical interventions are becoming more and more complex with a less wide operative field. In this sense, one should use new tools to achieve a safe radical anatomical resection.

Multi-detector CT able to construct three-dimensional (3D) images has been developed and adapted for clinical use over time in the field of thoracic surgery (12). 3D reconstruction of pulmonary vessels and the bronchial tree are increasingly used for preoperative and intraoperative virtual model (13), helping with the surgical performance.

## The contribution of virtual reality

If reality is philosophically described as the world that we taste with our senses, augmented reality refers to the integration of the actual world with digital information: virtual reality indicates a complete digital representation of the actual world and with mixed reality we introduce possible elements into the actual world (14). Technological advances applied in thoracic surgery allowed the integration of these “deformations” of reality in the surgical world. With the clinical application of the augmented reality immersive 3D-platforms, the surgeon’s arsenal is becoming increasingly vast, allowing to perform more complex procedures with a safer minimally invasive approach.

## Preoperative surgical planning

To best prepare a surgical intervention, the surgeon must carefully study the patient imaging such as computed tomography to recognize cancer localization and the surgical strategy to achieve a good resection with safe surgical margins. This concept is the basis for any surgeon who performs both open and minimally invasive surgery. The preoperative planning also serves to foresee possible difficulties and manage complications. In VATS, preparation becomes even more important since one has to deal with a limited and indirect surgical vision. A more accurate preoperative planning is mandatory for segmentectomies as the sublobar anatomy is subject to even more anatomical variations than the lobar anatomy, to avoid arterial or veinous injuries. It is helpful for surgeons to convert 2D CT information into 3D representations of the patient’s anatomy (virtual or printed models) and this becomes possible with image-guided technology to visualize lung anatomy in a 3D view, available before and during surgery. Several companies propose virtual 3D reconstruction imaging either by performing the service on-line or by selling software for surgeons to perform their own reconstruction.

## Intraoperative tools

We can divide the intraoperative tools into three main categories: those for the anatomical study of vessels and bronchi, those used for the localization of the cancer and those for the identification of segmental borders. All concern small pulmonary nodules, for which sublobar resection is recommended. The use of these tools could result in a reduction of operation time and conversion rate.

**Figure 1:**
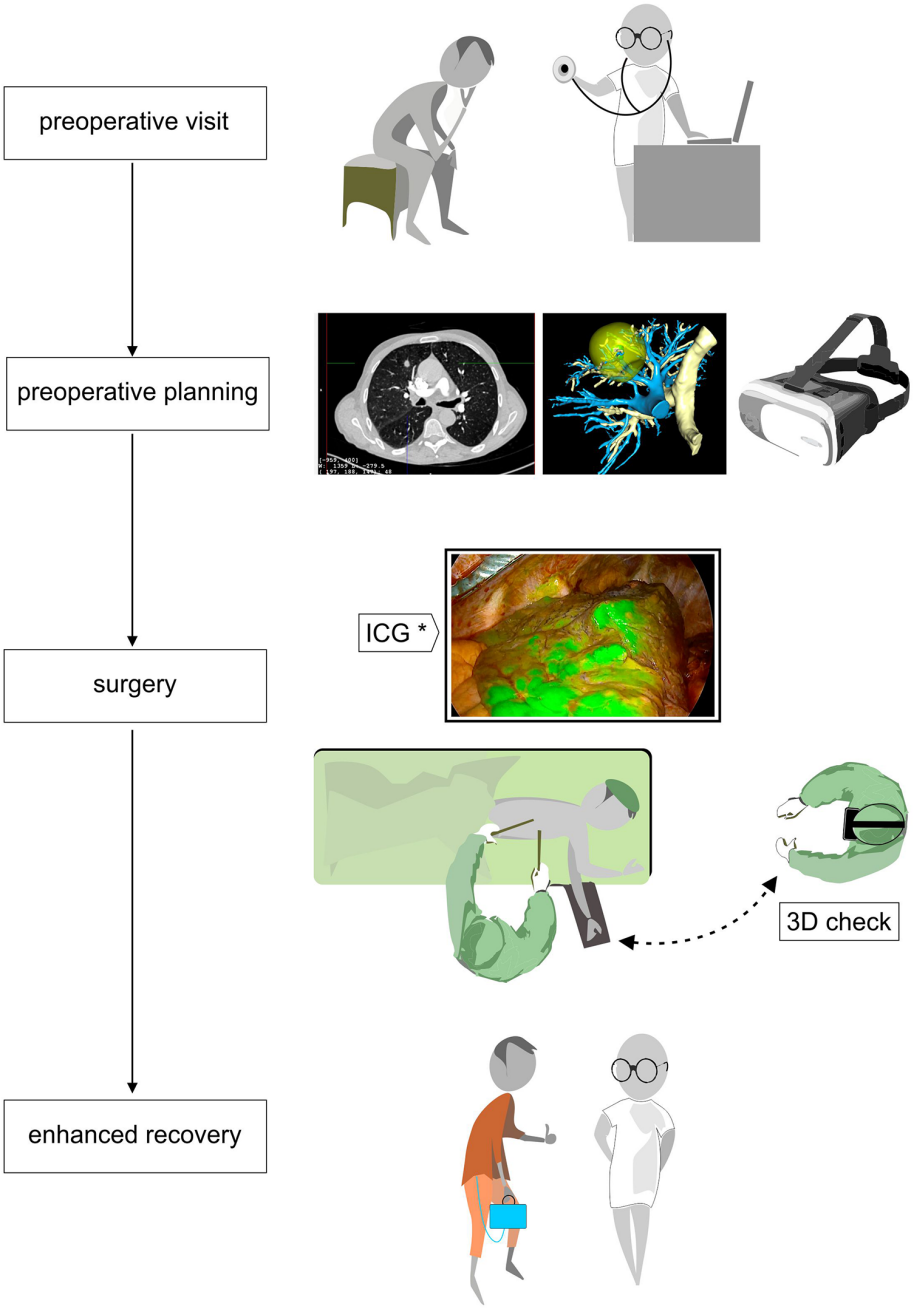
Flowchart of patient care from the preoperative visit with CT and 3D reconstruction in virtual or mixed reality by AI-based platform that enables preoperative planning; operative time with intraoperative tools like hololens to verify patient anatomy and infrared thoracoscopy with systemic ICG intravenous injection to define the ISP. Finally, the ERAS program with a digital drainage system.

In the operating room, surgical planning can be available on PC, tablets or smartphones to check for patient’s anatomy. More recently, the augmented reality has evolved to mixed reality thanks to the development of Hololens headset from Microsoft 3D modeling visualization (Microsoft Corporation, Redmond, Washington, USA) (Figure 1). The surgeon has the possibility to a completely rotate the holographic model and adapt it to the operative view. Certain companies also propose 3D-printed models that can be hold during surgery and even coupled to a augmented reality platform to facilitate complex thoracoscopic surgery (15).

During thoracoscopic segmentectomy, direct palpation of the tumor is not always possible, and achieving adequate tumor-free margins from the cancer is of crucial importance.

Regarding this difficulty of identifying and localize small nodules during surgery, several tools have been proposed in literature using augmented or mixed reality. New navigation-guided systems have been described as a safe and feasible technique. A pilot study by Li et al. was published showing an innovative marker (LungBrella marker) for small pulmonary nodules revealed during surgery by augmented reality software (16).

Another mixed-reality interesting tool proposed by Perkins et al. would allow a better understanding of the localization of the lesion with anatomical features of interest according to the degree of the lung deflation during surgery (17). The authors describe the possibility of simulating lung movement and even the placement of surgical instruments, to easily identify small lesions.

Regarding segmental borders, they can be determined by augmented reality 3D software so that resection margins can be determined preoperatively. Intraoperatively, the identification of the intersegmental plane (ISP) for segmentectomy may be very challenging. Infrared thoracoscopy with systemic ICG intravenous injection (Figure 1) after the division of the segmental blood vessels and bronchi showing fluorescence in all structures except the isolated segment to be resected (18), is an easy reliable method to guide segmental parenchymal resection. Other techniques have been proposed: injection of endobronchial dye via electromagnetic navigation and virtual-assisted lung mapping (19, 20), the use of 3D simulation using multidetector CT (21), and artificial intelligence-assisted mapping planning with a combined dimensional reduction method (22).

## Postoperative management

The concept of VATS is strictly linked to the notion of enhanced recovery after surgery (ERAS). ERAS pathways aim to reduce the effects of perioperatively induced stress response and focus on the quality of a patient’s recovery (23). In this perspective, an early mobilization of the patient is essential, and the use of portable continuous suction systems could be privileged (Figure 1). Digital drainage systems are now widely available: they are light, compact and have a built-in suction pump, improving clinical decision-making through continuous monitoring of air leaks and fluid loss. As we have an objective quantification of these leaks, drains can be safely taken out early.

## Augmented reality: toy or tool?

The interest for the surgeon is obvious. The application of technology and augmented reality has numerous and easily imaginable advantages in terms of best preparation of the intervention due to a complete comprehension and knowledge of patient’s anatomy, best planning, and intraoperative assistance. Arjomandi Rad et al. described the role of augmented reality in reducing preoperative planning time and workload, helping surgeons to perform complex procedures more safely by facilitating the understanding of patient-specific anatomy and anatomical variations (24). The use of a 3D reconstruction can provide a safer intervention, in terms of less intraoperative complications (e.g., vascular or parenchymal tear or inappropriate resection), reduced operative time and improved carcinologic margins (25). Creation of 3D printable models and virtual 3D reconstructions may also be used, not only as a teaching support for medical students and young residents, but also for the patient, to clarify surgery and the extent of the procedure. Using modern technology in VATS and the ERAS protocols can lower the risk of surgical intraoperative complications and postoperative complications (26), and, as a consequence, ambulatory or post-operative day1 discharge after pulmonary major resection surgery can become more realistic in suitable cases.

At our department we routinely use preoperative 3D reconstruction of pulmonary anatomy before performing VATS segmentectomies. We can study the anatomy and define a preoperative strategy by sharing it with our residents. During surgery we have the possibility to check the reconstructions via the application on smartphone and PC and occasionally we have access to mixed reality with the Hololens support.

## Pedagogical issues

### How to develop competence in VATS?

Current studies on the use of augmented reality in thoracic surgical training are limited to VATS, but are particularly beneficial for junior trainees, by allowing them to operate in a realistic, yet ethically risk-free setting to accelerate their learning process. Developing skills in VATS becomes an even more important concept for the surgeon since it is necessary to know how to manage the situation in a small space and with an indirect vision provided by the optics and with endoscopic instruments. On the other hand, the necessity of emergent conversion to thoracotomy in case of vascular complications can be overwhelming.

One of the best opportunities in VATS is the possibility to reproduce the main operations in a simulation platform to progress one’s personal learning curve in an uneventful way. The training course in surgery varies from country to country and from center to center based on the possibilities. Currently, there is no standardization in training for VATS and major pulmonary resections, such as lobectomy. Classically we go step by step starting with minor operations such as pleural biopsies, sympathectomies, pneumothorax to become familiar with the instruments and the 2D vision of the optics (27).

**Figure 2:**
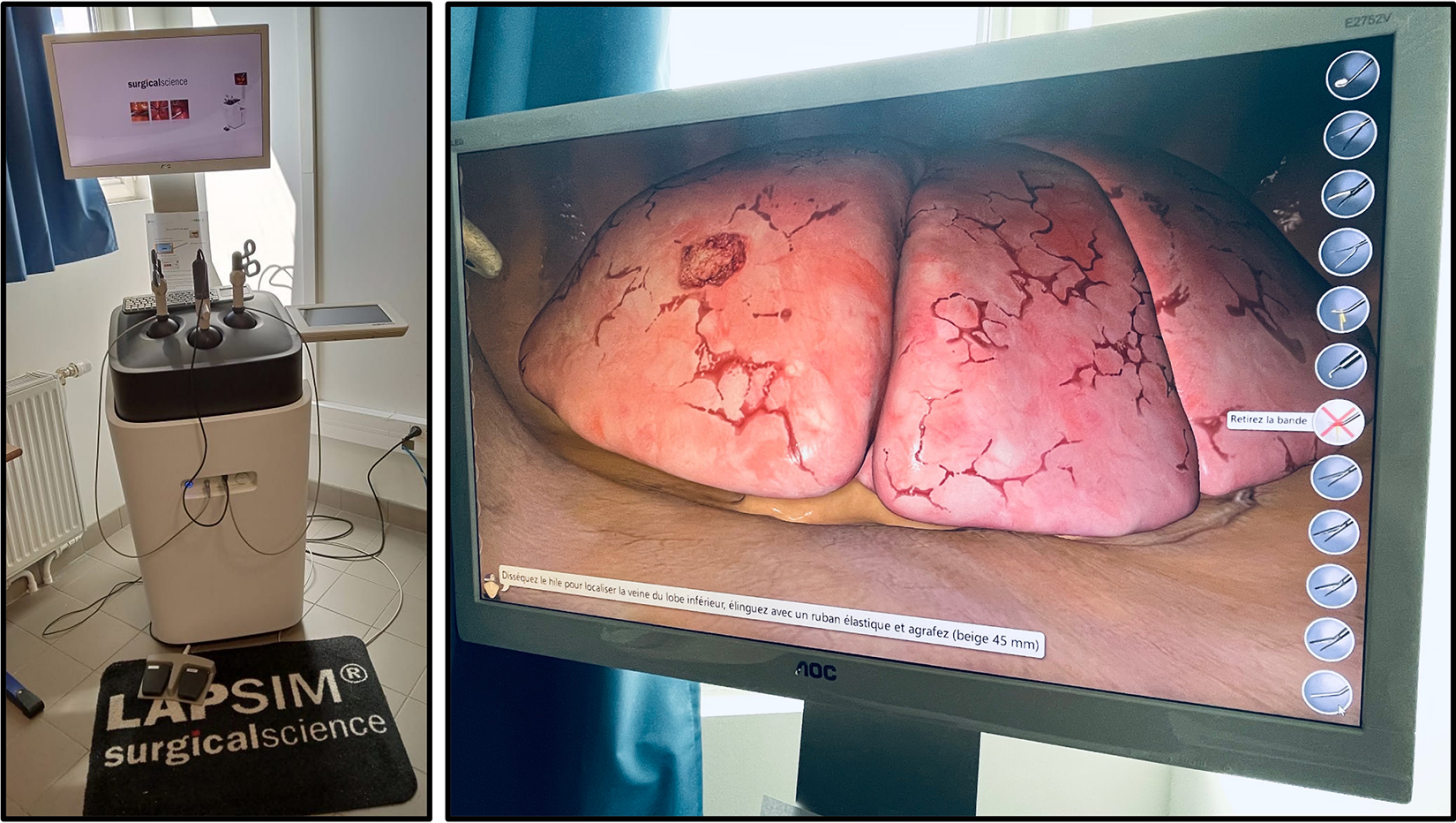
Lapsim™, surgical science, at the university of sorbonne nord, bobigny (France). Virtual reality simulation platform with thoracic surgery module enabling all five types of lobectomy.

The first virtual reality simulator for training VATS lobectomy (LapSim™, Surgical Science) (Figure 2) has shown promising results regarding realism and ability to discriminate between surgeons with different experiences (28). Konge et al. describe the use of a “four-step approach” to medical simulation programs starting with theoretical to hands-on training (28). A recent randomized controlled trial reporting the clinical impact of virtual reality training in laparoscopy has proved to increase the operative performance level of junior and less experienced surgeons (29). An international consensus sought to identify and prioritize technical procedures for simulation-based training to integrate into the thoracic surgical curriculum, the first of which were VATS lobectomy followed by VATS segmentectomy to meet current residents need (30).

At our university Sorbonne Paris Nord, we have free access to a LapSim™ station with training programs in thoracic and general surgery. We organize for each new group of residents’ simulation training. Once they are able to fully manipulate the instruments and perform their procedures (several lobectomies), they follow without supervision. In our simulation program, we train two residents at a time, one as a “main” operator performing the surgery and the second as an assistant (especially manipulating optics). We believe it is important that both learn to help and be helped during simulation as during “real” surgery.

## Conclusion

In the present time, using and making available adequate existing technology provides best preparation and improved surgical skills in VATS. It can also improve patients’ awareness of the procedure and their personal implication in the management of the postoperative period. Consequently, one can imagine less intraoperative and postoperative complications, both early and late term. Future development and use of simulators can promote VATS anatomical resection learning, standardize the learning curve and improve crisis management. As VATS surgery is constantly growing, it is important that simulation programs be integrated in the thoracic resident curriculum.
